# Analysis of the association between facial biotype, overbite and overjet in the permanent dentition

**DOI:** 10.4317/jced.60301

**Published:** 2023-05-01

**Authors:** Liz Chite-Quispe, Marco Sánchez-Tito

**Affiliations:** 1 Facultad de Ciencias de la Salud, Universidad Privada de Tacna, Tacna, Perú; 2 Specialist in Orthodontics and Maxillary Orthopedics

## Abstract

**Background:**

To determine the association between the facial biotype, the overbite and the overjet in digital lateral skull radiographs.

**Material and Methods:**

230 digital skull lateral radiographs of patients with permanent dentition with 18 to 55 years old were used as sample. The VERT index and the Ricketts analysis were considered to identify the facial biotype, overbite and overjet.

**Results:**

The facial biotype with the highest prevalence was dolichofacial with 36.52% (n=84), being more frequent in the female sex. However, the mesofacial biotype predominated in the male sex. On the other hand, the normal overbite presented the highest percentage with 66.08% (n=152) and the deep bite was more frequent than the open bite. Regarding the overjet, the normal overjet was the most prevalent with 68.70% (n=158), followed by the decreased overjet. Differences were found between the overbite values of males with brachyfacial biotype versus mesofacial and dolichofacial (*p*<0.05). A strong positive correlation was observed between overbite and overjet in mesofacial biotype (Rho=0.83, sig=<0.001). The predominant vertical malocclusion in the dolichofacial biotype was open bite and deep bite in the brachyfacial biotype. Regarding the facial biotype and the overjet, a significant association was found only in the male sex (*p*<0.05).

**Conclusions:**

With the results obtained, it is concluded that there is an association between facial biotype, overbite and overjet.

** Key words:**Facial biotype, overbite, ovejet.

## Introduction

During orthodontic treatment planning, it is necessary to evaluate different characteristics such as facial biotype, growth patterns, and intra- and inter-arch dental relationships (1,2). According to Ricketts, the facial biotype is the set of morphological and functional characters that determine the direction of growth and the functional behavior of an individual’s face (3).

The facial biotype may affect facial harmony, orofacial muscles, occlusion and dental position, aesthetics, and the function of the stomatognathic system (4-7). The facial biotype can be determined by different methods, some based on clinical examination, photographs and cephalometric analysis. Cephalometric standards such as the Ricketts VERT analysis, the Björk-Jarabak coefficient and the Downs-Steiner divergence of bone bases exist for this purpose (8,9).

Malocclusions reflect alterations in occlusion and craniofacial relations that can impair different aspects such as aesthetics, function, facial harmony and the psychosocial well-being of a person (10-12). According to Lombardo *et al*. the global prevalence of malocclusion in 2020 was 56%, being higher in Africa (81%), followed by Europe (72%), America (53%) and Asia (48%) (13).

The choice of treatment to follow will depend on several factors such as the severity of the skeletal anomaly or aesthetic and occlusal aspects, which can affect dental relationships such as overbite and overjet (14,15). Both occlusal relationships can be determined through clinical examination or through Ricketts analysis (16).

Several studies have reported the relationship between the facial biotype and the presence of malocclusions, mainly with sagittal malocclusions (17,18). Claro *et al*. (19) related the overbite and the craniofacial growth pattern and found no dependency relationship, that is, an increase in overbite could not be associated with a brachyfacial growth pattern, nor could open bite be associated with a dolichofacial growth pattern. On the other hand, Ioannidou *et al*. (20) studied the relationship between overbite, overbite and craniofacial morphology, concluding that the occlusal characteristics were not associated with any particular skeletal pattern, however a significant correlation was found between overbite and overjet.

Currently, there are no studies that relate facial biotype, overbite and overjet. For this reason, the main objective of the study was to determine the association between facial biotype, overbite and overjet in a sample of patients with permanent dentition requiring orthodontic treatment.

## Material and Methods

This research was approved under registration No. 117-2022-UPT/FACSA-D. The population consisted of digital lateral skull radiographs, taken from patients with permanent dentition, between 18 and 55 years of age, treated at the “El Galeno” Radiological Center, in the city of Tacna (Peru) during 2020-2022. The total record for that period was 2050 radiographs. The sample size was obtained through the comparison of proportions from a previous study (19). Considering a sample size of 230 digital lateral skull radiographs. The radiographs were randomly selected. The selection criteria were: X-rays of patients with complete permanent dentition, without distortion, without premature loss of teeth, without a history of orthognathic surgery, without marked dental wear, who are not undergoing orthodontic treatment and without coronary destruction of the teeth.

The radiographs were taken using the Orthophos SL 3D Ceph radiographic system (Dentsply Sirona, Germany). Operated at 85 kVp and 8 mA, with an exposure time of 14.18 s and a Voxel size of 80 µm. The digital cephalometric analysis was performed with the Nemoceph® program (Software Nemotec SL, Madrid, Spain), which allowed obtaining the VERT index and the Ricketts analysis. For the cephalometric tracing, some brightness and contrast filters were used to improve the visualization of the anatomical structures in the image. The fine adjustment of the points and curves was given using the Bezier curves tool. The cephalometric analysis was performed in a suitable and illuminated environment and by a single operator. All the data obtained were recorded in an observation sheet. The values for the identification of the facial biotype, overbite and overbite can be observed in Table 1.

**Table 1:**
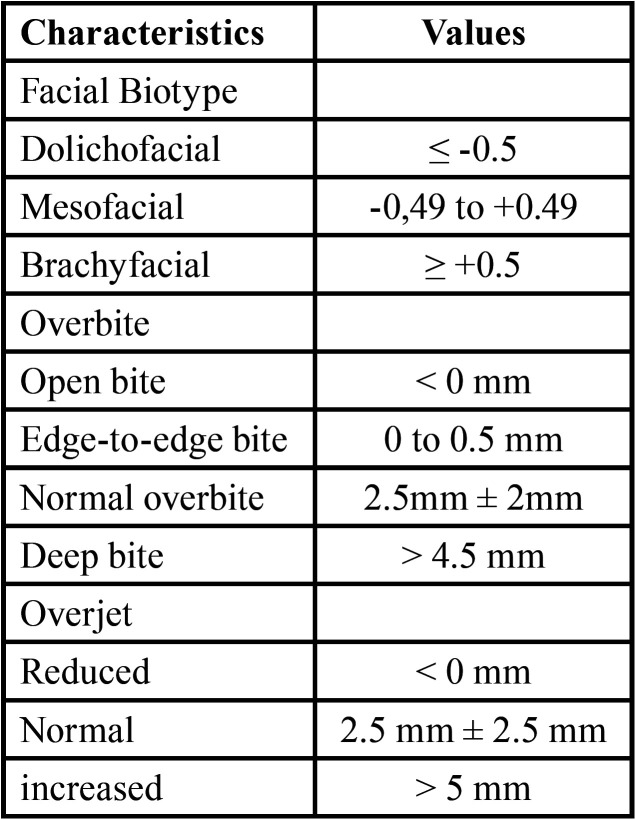
Values of the facial biotype, overbite and overbite.

To assess intra-examiner agreement, cephalometric measurements were performed on 23 lateral skull radiographs, which were randomly selected at two moments, with an interval of one week. To assess inter-examiner agreement, the cephalometric measurements obtained were compared with the expert’s measurements.

Data analysis was performed with the Stata® program version 15.0 for Windows. Descriptive and analytical statistics were used, such as the ANOVA and Student’s t test, to determine significant differences between the overbite and overjet values according to facial biotype and sex. The correlation between the overbite and the overbite was determined using Spearman’s coefficient and Fisher’s exact test was used to determine the association between the facial biotype, overbite and overbite according to sex. The significance level considered in this study was 5%.

## Results

The concordance was assessed through the intraclass correlation coefficient at a confidence interval of 95%, coefficients greater than 0.75 were obtained, indicating a good concordance on the intra-examiner and inter-examiner agreements.

The most prevalent facial biotype was dolichofacial with 36.52% (n=84), being more frequent in the female sex. However, the mesofacial biotype predominated in the male sex. On the other hand, the normal overbite presented the highest percentage with 66.08% (n=152) and the deep bite was more frequent than the open bite. Regarding the overjet, a normal overjet was the most prevalent with 68.70% (n=158), followed by the decreased overjet (Table 2).

**Table 2:**
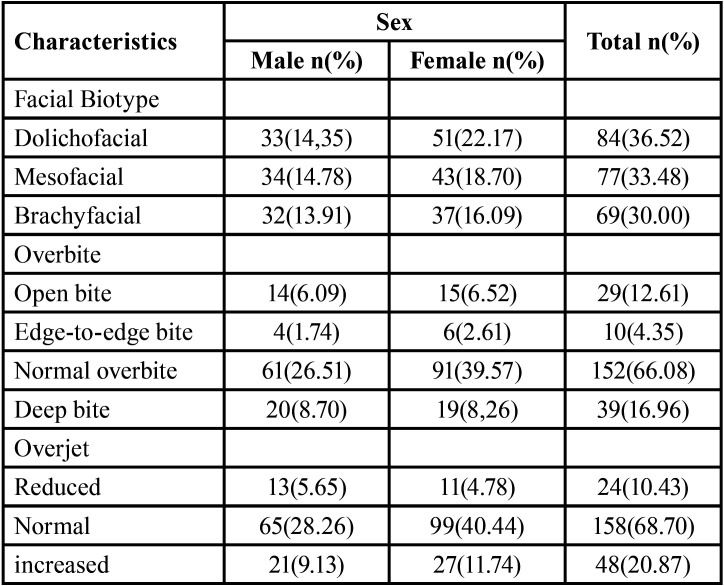
Frequency distribution of the facial biotype, overbite and overjet, according to sex.

**Figure 1:**
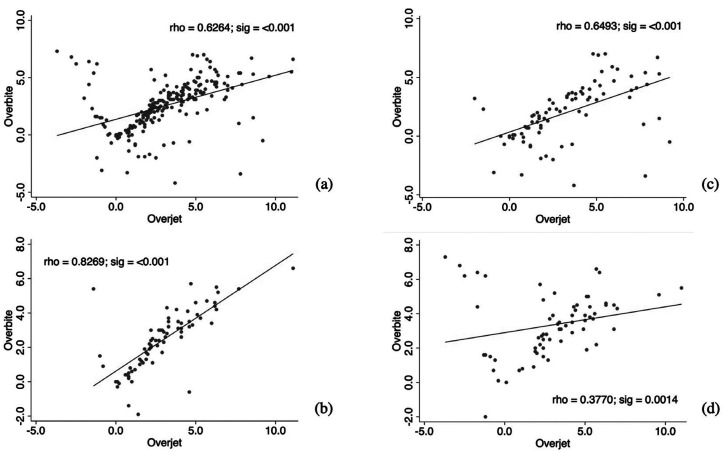
Correlation between overbite and overjet. (a) Moderate positive correlation between overbite and overjet in the total sample. (b) Strong positive correlation in the mesofacial biotype. (c) Moderate positive correlation in the dolichofacial biotype. (d) Weak positive correlation in the brachyfacial biotype.

Differences were found between the values of the overbite of males with brachyfacial biotypes versus mesofacial and dolichofacial (*p*<0.05). No significant differences were observed for the overjet between sex and facial biotype (Table 3). In addition, a strong positive correlation was observed between overbite and overjet in the mesofacial biotype (Rho = 0.83; sig = <0.001) (Fig. 1).

**Table 3:**
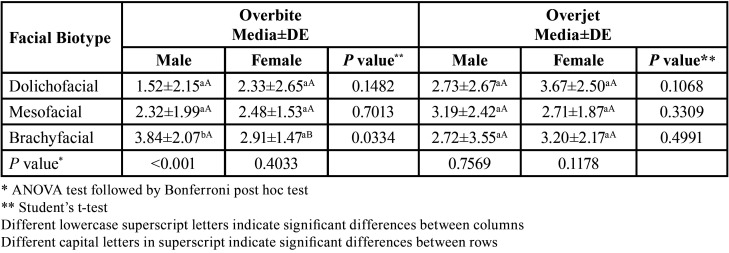
Differences between overbite and overjet values according to facial biotype and sex.

Table 4 shows that the predominant vertical malocclusion in the dolichofacial biotype was open bite, and deep bite in the brachyfacial biotype, finding a significant association between these two variables in both sexes (*p*<0.05). Regarding the facial biotype and the overjet, a significant association was found in the male sex (*p*<0.05) (Table 5).

**Table 4:**
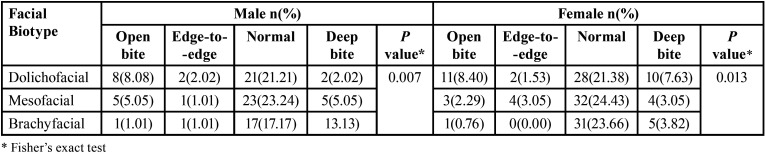
Association between facial biotype and overbite according to sex.

**Table 5:**
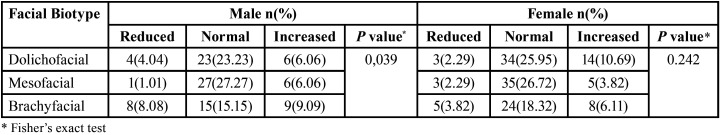
Association between the facial biotype and overjet according to sex.

## Discussion

The facial biotype is the set of morphological and functional characters that allows determining the direction of growth and behavior of the facial structure. The facial biotype has been widely studied over time, being associated with different variables such as gingival tissue (21), canine impaction (22), sagittal malocclusions (17), vertical malocclusions (19).

In the present study, the facial biotype was determined through the VERT index, the overbite and overjet through the Ricketts analysis. The Nemoceph ® program was used for the digital cephalometric analysis. This software has a high reliability, which provides values very similar to conventional tracing, being very precise, currently it is widely used for clinical diagnosis and provides very satisfactory results (23).

The VERT index is widely used to determine the facial biotype (24,25), either through the conventional method or with the use of specialized programs in cephalometric analysis. Likewise, it has been shown that there are no significant differences between the different programs, nor between the manual and digital cephalometric analysis (26,27). However, it should be considered that some cephalometric analyzes differ from each other, showing differences in the diagnosis, so performing alternative analyzes is a good option to make better decisions before carrying out orthodontic treatment (28). In this study, Ricketts’ analysis was used since it is the most widely used, it presents better diagnostic concordance with other analyzes and presents greater reliability when evaluating vertical growth.

The predominant facial biotype in this study was dolichofacial, followed by mesofacial and brachyfacial. These results are different from those reported by Assiri *et al*. (21), where it was found that the mesofacial biotype (41.2%) was the most common, followed by dolichofacial (37.5%) and brachyfacial (21.3%) to a lesser extent, in a population of Saudi Arabia. The results of Crincoli *et al*., (22) and Pacific *et al*. (7), observed a higher percentage of the mesofacial biotype (52%), followed by brachyfacial (32%) and dolichofacial (16%) in the Italian population. Costea *et al*. (4), reported the mesofacial biotype (50%) as the predominant one, followed by dolichofacial (26.56%) and brachyfacial (23.44%) in a Romanian population. Finally, Niño *et al*. (29), in their study in Brazil, observed that the mesofacial biotype (51.25%) was the most frequent, followed by dolichofacial (28.33%) and brachyfacial (20.42%).

In this study, it was observed that more than half of the sample presented normal values of overbite and overjet. The predominant vertical malocclusion for both sexes was deep bite, followed by open bite and edge-to-edge bite. In addition, the increased horizontal projection was presented in a higher percentage than the decreased one. These data coincide with the global distribution of malocclusion presented in the study by Alhammadi *et al*. (30), in which the different geographical areas of the world are considered. Significant differences were also evidenced between the values of the male and female overbite in the brachyfacial biotype, that is, the sex of a person could influence the presence of a vertical malocclusion, observing a sexual dimorphism.

Additionally, an association was found between the facial biotype and the overbite, in both sexes. Likewise, a significant association was found between the facial biotype and the overbite in the male sex and a positive correlation between the overbite and the overbite. Concerning the association between the facial biotype and the overbite, the results differ from what was mentioned in the study by Platou and Zachrisson (31), where it is stated that the brachyfacial have better occlusal relationships compared to the other facial biotypes, however in the results presented, the mesofacial are those that present these characteristics in the anterior occlusal plane. Regarding the correlation between the overbite and the horizontal overjet, we agree with the findings of Ioannidou *et al*. (20), in which that correlation was reported, with a coefficient of 0.27 according to Kendall’s Tau-b correlation.

Türkkahraman and Zayin (32), observed that certain skeletal facial characteristics are associated with certain alterations that affect overbite and overjet, such as anterior crowding. Likewise, Fattahi *et al*. (33), point out that counterclockwise mandible rotation, characteristic of the brachyfacial biotype, occurs in patients with a deep bite.

## Conclusions

Based on the results, we can conclude that there is an association between facial biotype, overbite, and overjet in the studied sample. This association is due to the morphological characteristics of the patients.
